# A Lightweight Method for Defense Graph Neural Networks Adversarial Attacks

**DOI:** 10.3390/e25010039

**Published:** 2022-12-25

**Authors:** Zhi Qiao, Zhenqiang Wu, Jiawang Chen, Ping’an Ren, Zhiliang Yu

**Affiliations:** 1 School of Computer Scinece, Shaanxi Normal University, Xi’an 710119, China; 2 Key Laboratory of Modern Teaching Technology, Ministry of Education, Xi’an 710062, China; 3 School of Mathematics and Computer Science, Shaanxi University of Technology, Hanzhong 723001, China

**Keywords:** graph data, defend, graph transformation

## Abstract

Graph neural network has been widely used in various fields in recent years. However, the appearance of an adversarial attack makes the reliability of the existing neural networks challenging in application. Premeditated attackers, can make very small perturbations to the data to fool the neural network to produce wrong results. These incorrect results can lead to disastrous consequences. So, how to defend against adversarial attacks has become an urgent research topic. Many researchers have tried to improve the model robustness directly or by using adversarial training to reduce the negative impact of an adversarial attack. However, the majority of the defense strategies currently in use are inextricably linked to the model-training process, which incurs significant running and memory space costs. We offer a lightweight and easy-to-implement approach that is based on graph transformation. Extensive experiments demonstrate that our approach has a similar defense effect (with accuracy rate returns of nearly 80%) as existing methods and only uses 10% of their run time when defending against adversarial attacks on GCN (graph convolutional neural networks).

## 1. Introduction

**Figure 1:**
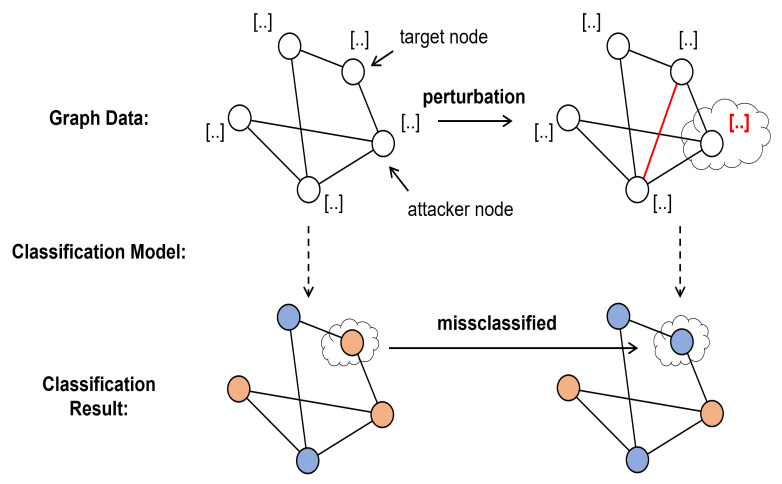
Adversarial attack on graph data (evasion attack).

Graph data processing is a problem in many fields such as e-commerce [1], social networking [2], and traffic [3]. A popular model for extracting features from graphs is the graph neural network [4], which performs node classification [5,6] and link prediction tasks with a high degree of accuracy. GCN [7], a kind of graph neural network with very balanced indexes, has lately been widely employed in node tasks including traffic forecasting, pharmaceutical design, and recommender systems. However, several studies suggest that Graph neural networks, including GCN, are susceptible to adversarial attacks as other neural networks. Some attackers can even manipulate the results of graph neural networks [8]. Figure 1 shows a form of adversarial attack on graph data. This is very disadvantageous to the practical application of a graph neural network. Thus, learning how to defend against adversarial attacks is vital and necessary.

Research in the field of defense has yielded some results. These strategies can be roughly divided into two broad categories. One of them is adversarial training. It usually makes some small perturbations specially in the training process, and then constantly adjusts the neural network to adapt to these perturbations to improve the robustness of the model. Dai et al. [9] proposed an adversarial training method suitable for graph embedding. The other kind of strategy is to design a robust graph representation learning method directly. R-GCN [10] uses this kind of strategy. These methods can effectively reduce the negative impact of adversarial attacks. However, in adversarial training, we need to use new datasets for adversarial training and get a more robust model to replace existing one. It is the same as using a robust strategy model. As we all know, in machine learning, it takes a lot of time to train a model. In practice, replacing the model may also mean other engineering problems such as interface and performance issues.

Here we propose a novel defense strategy. It can defend against adversarial attacks without training a new GNN model. Specifically, we want to use the characteristics of the complex network itself to build defenses rather than at the model stage. The motivation was that we found that a defense strategy directly oriented to the picture data performed well in the study of the attack and defense against the image adversarial samples. For example: padding or random resizing [11] an image adversarial example can fend off various adversarial attacks. Here, we call this the randomization approach. The randomization approach produces a superior protection impact with a very cheap computing cost in the application of picture data.

Another research also shows that low-level picture transformation [12] can disassemble the hazardous disturbances. At the same time, many adversarial attack strategies used in graph data come from research on picture data, including FGA [13]. The above two points enlighten us a lot: in graph adversarial attacks, modifying the edges, nodes, or properties of nodes in the graph is an inevitable process, just like modifying pixels in pictures. In the process of application and research of graph data, there are also many operations used to change graph structure, including topology and attributes. In the graph adversarial examples, a number of damaging structures may also be destroyed by operation, i.e., perturbing specific elements (nodes, edges, or node attributes). Here we call “operations” as “transformation”, collectively. The transformation approach also has the benefit of not requiring a separate training procedure.

### 1.1. Advantages

(1) The graph transformation method is model-independent and can be assembled before any graph representation learning algorithms. (2) This method does not require an extra training process so it requires low computational power. It also means users do not need to train new models to replace the working models.

### 1.2. Challenges

Graphs differ greatly from pictures in many ways. The arrangement of nodes and edges in graph is not as neat as the pixels in the picture. Therefore, we need to seek for procedures that may modify the graph without materially changing its current structure. At the same time, we also need to consider that the transformed graph data can still adapt to the input layer of the neural network.

### 1.3. Contributions

In this research, we present a lightweight defense graph neural network adversarial attack scheme with graph transformation technique. It is very suitable for the adversarial defense work under the time limit and insufficient equipment computing power. We introduce the implementation process of four transformation forms. After that, we use GCN to investigate the efficacy of the transforming method in graph data to defend against adversarial attacks. Finally, we analyze the experimental results and discuss more about transformation techniques. The contributions of this study are highlighted here:

- The transformation method optimizes the flow of defense against an attack on graph data. Our approach avoids extra training processes and achieves a shorter run time;
- We explore four graph transformations: copying, padding, deleting edges, and nodes compression. We also introduce how to implement these four transformation forms and their complexity is analyzed;
- A series of contrast experiments are designed, which cover both defense effectiveness and run time.

The remainder of this essay is structured as follows: in Section 2, we introduce relevant works. The background information, such as graph neural networks, adversarial attack, and defense strategies, are organized in Section 3. We discuss our methodology in Section 4. In Section 5, the experiments and their outcomes will be presented. We conclude by summarizing the entire essay and providing further work in Section 6.

## 2. Related Work

### 2.1. Adversarial Attack on Graph

The adversarial attack damage graph representation learning was first introduced by Zügner et al. [14] in 2018. They presented some concepts and the necessary parameters in the process of graph adversarial attack and defense, and developed the idea of modification cost, which prevents attackers from significantly altering graph data. In order to verify the possible effect of adversarial attack on graph data research, they also provided an iterative attack on GCN, called NETTACK. Wang et al. [15] proposed that it was unrealistic to attack the existing structure in graph data. They attacked graph data by maliciously adding harmful nodes and connecting harmful nodes to existing nodes. In that paper, a greedy algorithm was proposed to generate edges and the corresponding features of malicious nodes to minimize the classification accuracy of target nodes. Finkelshtein et al. [16] stated that attackers can only control a small number of nodes in practical cases. So, they proposed a method to attack the graph by perturbing only one node. They generated harmful nodes and propagated the malicious attributes of the harmful nodes to the target nodes through feature aggregation. Dai et al. [17] proposed an attack-method based on reinforcement learning, named RL-S2V, to learn the generalized attack strategy, that only needs the predictive class markers of the target classifier. After several years of development, the research of a graph neural network adversarial attack has been developed well and researchers have summed up the existing types of attacks and have categorized them as follows:

- Classification based on attacker knowledge: If the attacker is familiar with the data and parameters of neural network model utilized by the victim. This is referred to as a white box attack. White box attacks are virtually impossible to execute in the real world, but their influence is the most difficult to eradicate. A grey box attack, is when the attacker can get the data but not details of the model’s contents. NETTACK, mentioned above, is a kind of gray box attack. In a black box attack, an attacker can only obtain query results. A black box attack is the most concealed of the three attacks, so it is more likely to cause serious consequences. RL-S2V is the first time that a black box attack has been implemented in graph data;
- In machine learning, the attack can be classified as Poisoning or Evasion [18]. The poisoning attack happens before training and alters the training data. In general, poisoning attacks induce model failure. Although an evasion attack corrupts the test data and provides adversarial instances, the victim model remains intact.

The above classification includes all the possible forms of an adversarial attack on graphs at present, and also provides a foothold for studying how to defend against an adversarial attack. We will also use all types of attack methods to evaluate the defense methods by experiments.

### 2.2. The Defense Approach

Adversarial defense research is advancing quickly with the creation of attacking graph neural networks. Feng et al. [19] consider that a node has a high probability of being correctly classified when it has the same classification as its neighboring nodes. They designed a regularizer, which can generate a similar node to those of its neighbors. Zhou et al. [20] proposed a new method to accurately measure the possibility of existence of queried links. They define the concepts of an analyst (defender) and an attacker, and model the interaction between analyst and attacker as a non-zero-sum Bayes Stackelberg game [21]. Zhu et al. [10] proposed a robust graph neural network with attention mechanism [22,23], R-GCN uses Gaussian distribution instead of a feature vector as the hidden representation of nodes in each convolution layer. When the graph is attacked, R-GCN can automatically absorb the adverse effects of variance changes of the Gaussian distribution. This kind of idea has influenced recent studies to further focus on proposing more robust graph neural network models. Raghu Arghal et al. [24] used Probabilistic Lipschitz Constraints to improve robust and Chen et al. [25], focus on node aggregation and replaced the mean value with median value in aggregation and proposed a more reliable graph convolution model.

As these new methods are proposed, defenses are becoming better and better. However, they all inevitably require lengthy and expensive training sessions. As we can see, an adversarial training method includes a long training process during the application process, while using more robust models also requires training new model to replace the old one.

Is there a class of methods that do not require any additional training process? Finally, we were inspired by the field of image adversarial defense. Xie et al. [12] used random transformations to decrease the effects of an adversarial attack. They transformed the size of images and padded zeros pixels around resized images randomly before inputting them into neural networks. We found that the randomization method works at the input data stage and is weakly associated with the model, so this work can be completed on the basis of an invariable model. Based on this idea, many defense methods based on modified input data have emerged in the field of an image anti-attack: Guo et al. [26] extended this kind of method. They extended this method [27] with JPEG [28] (Joint Photographic Exports Group) compression and image quilting. JPEG is a type of lossy compression with discrete cosine transform. It can cause transformations to the pixels that are imperceptible to the naked eye. These studies are all based on images and we do not see any idea similar to them in the graph. Some researches use Gaussian data enhancement in an image to defend against attacks [29].

The defense method of modifying input data directly provides a new idea and its availability and effectiveness has been verified in the image field. As far as we know, no studies have applied this idea for graph data to reduce the impact of adversarial attacks on GNN models.

## 3. Preliminaries

Here we firstly define some key concepts and provide brief explanations of the notations we will be using throughout the work.

### 3.1. Definition

In this study, a graph is defined as G=(V,E,X), where *V* represents the set of nodes, $V=\left\{v_{1},v_{2},\ldots ,v_{N}\right\}$ and N=|V| denote the number of nodes. *E* represent the set of edges and E⊆V×V. *X* indicates the attribute of the nodes, which is a N×D matrix. In reality, a node’s attribute is the amount of its quantified characteristic or the categorization of a characteristic. A person’s degree of support for a proposal, for example, can be measured as 0, 1, or 2. Here, 0 indicates strong opposition, 1 indicates fairness, and 2 indicates strong support. One node’s attribute can be denoted as $x_{i}=\left(x_{i1},x_{i2},\ldots ,x_{iD}\right)$. In our method, the adjacency matrix of the graph is used to participate in the operation. An adjacency matrix is represented as *A*, which is a N×N matrix. For the node classification task, the label of the nodes is *C*. *C* is an 1×N matrix as $C=\left\{1,2,\ldots ,c_{N}\right\}$ and $c_{i}$ represents the label of a node in the dataset. A classifier *f* with a series of parameters θ (sometimes θ will be omitted for brevity). At the same time, we input *G* to the classifier with *C* as the truth label. The classification process can be represented as:

(1) $$\begin{array}{cc} f_{\theta } & :G\to C^{\prime }\\ \; & C^{\prime }\approx C, \end{array}$$

where $C^{\prime }$ is the predicted labels of the classifier *f*. A good classifier will make the prediction labels very close to the true label. However, a graph adversarial attack aims to make a small disturbance of input graph G and the contaminated data input will lead to obvious deviation of the classification results, which can be represented as: $\mathrm{attack}\left(G\right)=G^{*}$. This contaminated data input to the classifier will lead to obvious deviation between the predicted labels and the real labels: $f_{\theta }:G^{*}\to C^{{*}^{\prime }}$, and an attacker expects the distance between $C^{{*}^{\prime }}$ and *C* to be as far as possible. Oppositely, a defense method needs to minimize the negative effects of the adversarial examples:

(2) $$\begin{array}{cc} \underset{F}{\mathrm{argmin}} & \left(\mathrm{distance}\left(F\left(G^{*}\right),C\right)\right)\\ \; & F\left(G^{*}\right)=C^{\prime \prime }\\ \; & C^{\prime \prime }\approx C, \end{array}$$

*F* is for adversarial defense. Prior works consider adversarial training, which that means a new classifier needs to be trained: $f_{\phi }\neq f_{\theta }$. However, such approaches require training new model parameters with a high cost. In our scheme, we will keep the original model but transform the data input to the model:

(3) $$\begin{array}{cc} \mathrm{transform}(G^{*}) & =G^{**}\\ f_{\theta }:G^{**}\to & C^{\prime }\\ C^{\prime }\approx C, \end{array}$$

Our defense approach is independent of the model as a module, which makes our approach very portable. It also avoids training and saves computing power.

### 3.2. Target Model

The target model is the basis on which we measure effectiveness adversarial attack and defense. An experiment will be designed to compare the classification performance of the target model in its original state, after being attacked, and after the intervention of defensive measures.

We chose the GCN model as the experimental target, and finally used the accuracy of node classification as the evaluation index. The input to the whole process is a graph G=(A,X), where $A\in {\left\{0,1\right\}}^{N\times N}$ is the adjacency matrix and $X\in R^{N\times D}$ represent the features of nodes. The graph also has two kinds of basic elements: edges and nodes. Here G=(V,E), *V* means the nodes’ set and *E* for edges’ set. Each node *v* has a D-dim. feature vector as $x_{v}\in R^{1\times D}$. In a node classification task, each node has labels from $C=\left\{1,2,\ldots ,c_{k}\right\}$. The aim of node classification is to find a map $f:V_{train}\to C$ that maps each node $v\in V_{train}$ to one label in *C*. In this work, we consider using GCN to complete the node classification. In general, the GCN can be represented as

(4) $$H^{\left(l+1\right)}=\mathrm{ReLU}\left(\hat{A}X^{\left(l\right)}W^{\left(l\right)}\right),$$

(5) $$\hat{A}=D^{-\frac{1}{2}}\left(A+I\right)D^{-\frac{1}{2}},$$

where *A* is the adjacency matrix of graph *G* and *D* is the degree matrix of the graph *G*. *I* is the identity matrix that is used here to implement self-loops on undirected graphs. In the first layer of GCN, we have $H^{\left(0\right)}=X$. Here, we use a two-layer GCN to do the node classification task, represented as:

(6) $$Z=\mathrm{softmax}(\hat{A}\mathrm{ReLU}\left(\hat{A}XW^{\left(1\right)}\right)W^{\left(2\right)}),$$

and the result $Z_{vc}$ denotes the probability that a node *v* is classified as *c*. Although GCN is an efficient nodes classier, existing works show that GCN is vulnerable to adversarial attacks.

### 3.3. Producing Adversarial Examples

We consider three state-of-the-art adversarial attack models (NETTACK, FGA, RL-S2V) (1) NETTACK: One of the most classical graph neural networks against attacks and it is an iterative, local approach to attack. It builds a surrogate model:

(7) $$\log Z^{\prime }={\hat{A}}^{2}XW^{\prime },$$

The surrogate model of this approach removes the non linear function of GCN and preserves the process of graph convolution. They divide the attack process into structural and characteristic. The two attack processes are shown:

(8) $$\max_{\hat{A}}L^{\prime }\left(\log Z_{v}^{\prime }\right)\;\mathrm{where}\;\log Z_{v}^{\prime }={\left[{\hat{A}}^{2}W\right]}_{v}$$

(9) $$\max_{X}L^{\prime }\left(\log Z_{v}^{\prime }\right)\;\mathrm{where}\;\log Z_{v}^{\prime }={\left[W_{1}XW_{2}\right]}_{v}$$

(10) $$L\left(A,X;W,v_{0}\right)=\max_{c\neq c_{old}}{\left[{\hat{A}}^{2}XW\right]}_{v_{0}c}-{\left[{\hat{A}}^{2}XW\right]}_{v_{0}c_{old}}.$$

The attack iteratively looks for modification (structural or characteristic) that causes maximum loss to the surrogate model. NETTACK is local attack method that modifies some nodes to affect specific nodes. (2) FGA [13]: a kind of white box attack method. This method is based on the iterative gradient information obtained by trained GCN to generate an adversarial example. The author considers that the state of edge connection leads to gradient ascent. They focus on the loss function of GCN:

(11) $$L=-\sum_{i=1}^{|V_{train}|}\sum_{j=1}^{\left|C\right|}c_{ij}\ln\left(Z_{\mathrm{ij}}\right),$$

and take the partial derivative of it:

(12) $$g_{ij}=\frac{\partial L}{\partial A_{ij}}.$$

Now the target is to maximize the loss function L with gradients. However, graph data is discrete, so a gradient ascent cannot be directly used to maximize losses. Here the authors raise that if the connections state changes in the same direction as the gradient, the value of the loss function will increase at the fastest speed, and will symmetrize the gradient:

(13) $${\hat{g}}_{ij}={\hat{g}}_{ji}=\left\{\begin{array}{c} \frac{g_{ij}+g_{ji}}{2}\;i\neq j\\ 0\;i=j \end{array}\right.$$

The authors regard each $\hat{g}$ as a link gradient in the graph data. If a ${\hat{g}}_{ij}$ is positive/negative, it indicates that appending/removing a link between node $v_{i}$ and $v_{j}$ will increase the loss. In addition, the larger gradient ${\hat{g}}_{ij}$ along with the added link between $v_{i}$ and $v_{j}$ otherwise deletes the link. FGA is a kind of white box adversarial attack method. White box attacks are hard to implement in reality because models are usually transparent to users in real-world applications. It is almost impossible for ordinary attackers to get the details of the model. However, white box attacks can target weaknesses in the model and cause devastating damage. Therefore, it is significant to defend against white box attacks in theoretical research. (3) RL-S2V: This is a kind of black box attack. It needs the input original graph *G*, true label *C*, classification results $C^{\prime }$, and victim model *f*. RL-S2V regards the decision process of the adversarial attack as a finite Markov decision. It defines states and actions as follows:

- Action: $a_{t}$ means an attacker adds or deletes edges at time *t*;
- State: It uses a tuple $s_{t}=\left(G_{t},c\right)$ to present the state at time *t* and $\hat{G_{t}}$ represents the changed graph;
- Reward: the attacker’s goal is to interfere with the predicted results of the target classifier, so the reward is always zero except for when time is at the end of the decision process. If the prediction is consistent with the true label, this attack fails and returns −1 as the negative feedback. Otherwise, it returns 1 as the positive feedback.
  (14) $$r\left(\left(G,c^{\prime }\right)\right)=\left\{\begin{array}{cc} \; & 1:f(G,c^{\prime })\neq c\\ - & 1:\;f(G,c^{\prime })=c \end{array}\right.$$

In conclusion, the whole Markov decision process can be expressed as:

(15) $$(s_{1},a_{1},r_{1},\ldots s_{n},a_{n},r_{n},s_{n+1}),$$

and $s_{1}$ is original graph data with true label (G,c). Graph in state $s_{n+1}$ is the attacked graph $G^{*}$. Afterwards, the model uses Q-learning to learn this decision process:

(16) $$Q^{*}\left(s_{t},a_{t}\right)=r\left(s_{t},a_{t}\right)+\gamma \max_{a^{\prime }}Q^{*}\left(s_{t+1},a^{\prime }\right).$$

The first term on the right of the equation represents the reward feedback generated by the action of $a_{t}$ taken in the state of $s_{t}$ at time *t*. The second terms represents the maximum mathematical expectation of the reward that the following action can bring when the state changes from $s_{t}$ to $s_{t+1}$ after the action $a_{t}$ is taken. For more details of these methods please read the original article.

## 4. Transformation Method

**Figure 2:**
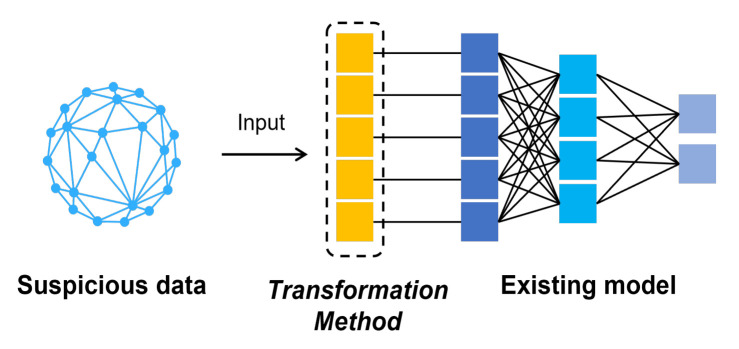
The process of the transformation method.

We introduce the transformation method in defending graph data adversarial attack in this part. Figure 2 shows the role of the transformation method in the defense process. The method’s analysis and details are as follows.

### 4.1. Execute Solution

#### 4.1.1. Nodes Compression

As previously stated, lossy image compression can accomplish adversarial defense. Can lossy compression of graphs do the job? A literature survey conducted by us showed that many graph compression techniques have been suggested in recent years. However, all of these solutions center around the web graph. These works were created using URL lexicographic order and a variety of encoding approaches [30]. Their primary purpose is to guarantee that graph query operations are successful. This method is very different from what we desire. Ref. [31] contains examples of such procedures.

**Figure 3:**
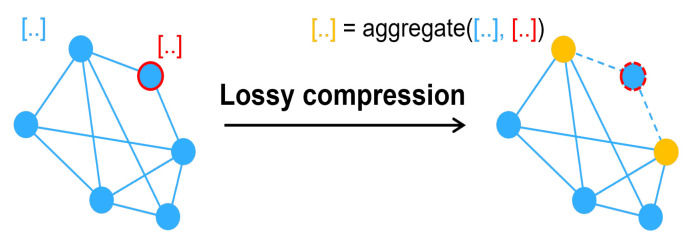
The process of node compression.

In order to achieve our goals, we create a unique graph data lossy compression technique named “nodes compression” as Figure 3. In most scenarios, such as in social networks and citation networks, the influence of low degree nodes on the graph is less than that of higher degree nodes [32]. Therefore, reducing the number of low degree nodes can preserve the main information of the graph and reduce the scale of the graph. However, it should be noted that low-degree nodes may have an abnormally high influence in some special networks. In this case, our compression method may not be suitable. This function iterates with the node degree. First we choose all nodes with a degree of 1 in the first iteration. Their attributes are aggregated (the method of aggregation is described below) with their neighbors’. We then remove the nodes with a degree of 1. If there are no more nodes of degree 1 in the graph, the node with the higher degree should be compressed. Of course, in addition to topology compression, there are also attributes that need to be compressed. This compression method will aggregate the attributes of the low degree node into the attributes of the adjacent and higher degree node. In this way, after the low degree node is deleted, its attributes will be included in the adjacent height node. Here we chose graph convolution as the aggregation method, and the aggregated attributes are assessed as follows:

(17) $$F=D^{-\frac{1}{2}}\left(A+I\right)D^{-\frac{1}{2}}.$$

Graph convolution collects node properties and graph topology. The aggregation procedure with node degree 1 is also taken into account. In this case, node compression is utilized not only as a stand-alone defense mechanism, but also as a necessary step in other transformation methods to maintain the size of the graph data the same as the original. Maintaining a consistent data size is essential in some graph neural networks, such as GCN.

The time complexity of this method is O(nlog(n)) and the memory complexity is $O(n^{2})$.

#### 4.1.2. Copy

**Figure 4:**
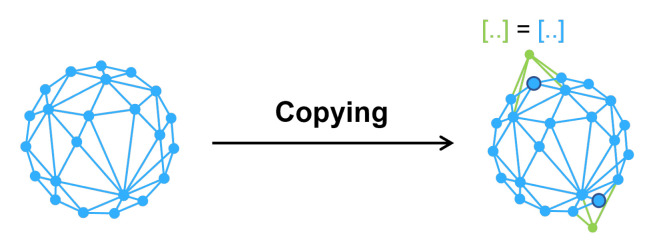
The process of copying nodes.

This idea is a random copy layer to classifier, which adds a number of copies of the existing nodes. This process is shown in Figure 4. $V=\left\{v_{1},v_{2}\ldots v_{m}\right\}$ is the original set of nodes. $V_{copy}$ is the set of newly added nodes and $V_{copy}\subset V$. The method goes through this set and randomly picks some nodes. We then copy the complete information about these node, including structure and feature. The number of nodes to be picked is another problem. According to our experiments, up to copy 5% of the number of nodes in the original graph data will get better results. For compatibility with efficiency and storage space, we recommend copying approximately 2% of the number of total nodes. This method has very good time complexity and O(n) at worst. The amount of extra storage required depends on how the graph data is stored. When the graph data is an adjacency matrix, the extra memory space required is $O(n^{2})$, while when the graph data is a set of edges the extra memory space needed is O(n).

#### 4.1.3. Pad

**Figure 5:**
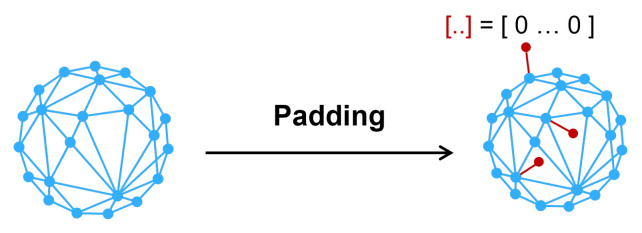
The process of nodes padding.

Another way to perform transformation is called padding, which adds some new nodes as Figure 5. Each of these newly added nodes has only one neighbor in the graph, i.e., which has a degree of 1. At the same time, each component of their attribute vector is 0. $V_{padding}=\left\{v_{p1},v_{p2}\ldots v_{pn}\right\}$, and for each $v\in V_{padding}$, $x_{v}=\vec{0}$. We call these newly padded nodes with attributes of 0 as “empty nodes”. Compared with the random copying method, the random padding method has more influence on node attributes. Similarly, experiments show that randomly padding nodes has relatively good effects between 1% and 5% of the number of nodes. We recommend padding 2% of the total nodes.

When we implement padding, we first choose a collection of nodes at random. Then, one by one, we add empty nodes to the current graph and match them with random nodes. The time complexity and memory cost of this padding technique are O(n).

#### 4.1.4. Delete Edges

**Figure 6:**
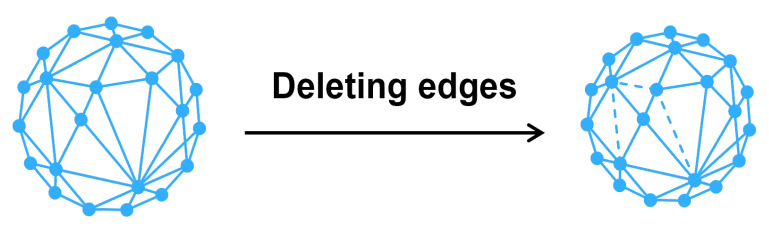
The process of randomly deleting edges.

Both approaches add new nodes to the original data. However, the additional data may have an adverse effect on very large scale graph data, such as running out of memory space. As a result, a graph transformation approach for ”subtraction” was offered. This method is summed up as removing edges, as Figure 6 shows. Edges are critical linkages that reflect the node relationships. The topological link between the two nodes is dissolved when an edge is eliminated. In actuality, it means that two items are physically separated from one another. This change impacts both the structure of the graph and the node properties. In order to implement this method, we turn the adjacency matrix into a collection of edges and delete some edges from it:

(18) $$\begin{array}{cc} \; & E=\left\{e_{1},e_{2},\ldots e_{n}\right\}\\ \; & E_{delete}=\left\{e_{d1},e_{d2},\ldots ,e_{dm}\right\}\\ \; & E_{delete}\subset E,E^{\prime }=E-E_{delete}. \end{array}$$

*E* is the set of edges, and we then pick some edges from *E* and delete them. The rest of the edges are $E^{\prime }$. In order to prevent excessive modification, the smaller one from the total number of edges and the total number of nodes is selected as the base to determine the number of edges to be deleted. The time spent on this process is O(n) and the extra memory space required is constant.

These three methods (copying, padding, and deleting edges) are still based on random selection. These three methods, however, are possible types of transformation procedures. Although the current experimental results show that the method based on random selection can also effectively play the effect of defense and counterattack, we believe that targeted selection of the transformed targets is more complete. This will be one of the points of focus of our following research. We also expect to have an additional discussion on the issue here. However, it is important to note that the current approach may not perform the best but it costs the least.

### 4.2. Analysis

All of the four approaches described above may be inserted as modules before the graph neural network’s input layer. At the same time, these specific methods can be mixed to achieve better defense. During the application process, for example, the input dimension of the trained GCN model is determined. However, some of random transformation methods may lead to an increase in the number of nodes, which does not match the existing model. In this case, a node compression can be performed before random transformation to cut some nodes and this keeps the dimensionality of the data unchanged. Considering the above situation, the time complexity of our method in the worst case is O(nlog(n)) and the maximum memory usage is $O(n^{2})$. Table 1 is a simple comparison of our approach with some existing strategies.

**Figure 7:**
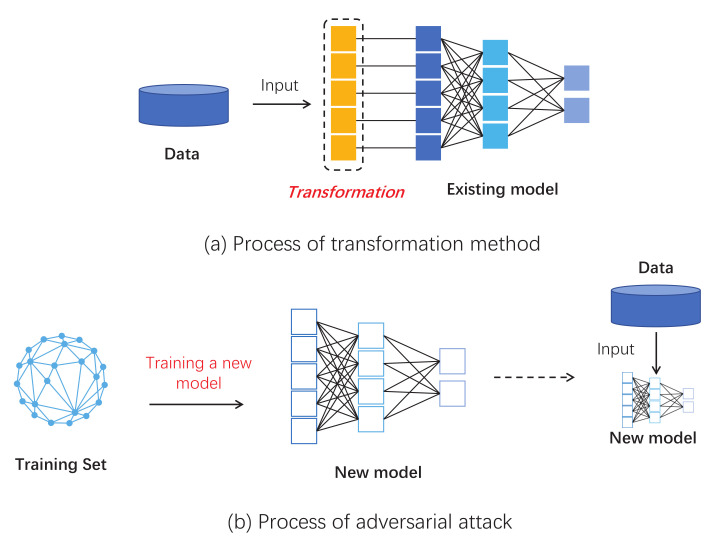
Comparison of the workflow of transformation and an adversarial attack. The transformation method can directly reuse existing models to process data, but adversarial training requires retrain a new model to replace the existing one.

Compared with the existing methods, graph transformation focuses on transforming data directly and improve the existing models in the face of an adversarial attack. No additional training is required. Therefore, our transformation methods are much faster than the existing defense methods based on adversarial training and robust models. Figure 7 shows the workflow advantages of the transformation approach.

## 5. Experiment and Discussions

In this section, we conduct experiments to evaluate the efficacy of the proposed approach. This section start by explaining the experiment’s circumstances and settings. Finally, the experimental data will be analyzed and discussed.

### 5.1. Dataset

Two graph datasets were selected for the experiment: Pubmed [33] and Cora [34]. These are real world datasets commonly used in graph neural network research. The Pubmed is a large-scale dataset. It comes from a well-known database in the medical field. The original content of the data was 19,717 scientific publications on diabetes and their citation relationships. Meanwhile 500 keywords were selected to form attributes in the dataset. These publications were eventually labeled into three categories, two different types of diabetes and diabetes related experiments. Cora is often used in the field of graph data science. The Cora dataset consists of machine learning papers, which are labeled in 8 categories. The network in Cora is also the composed of reference relation of the paper, with a total of 5429 reference relation. Table 2 shows more information about the two datasets.

### 5.2. Baseline Models

We choose GCN as the target model, which is a classical and the most commonly used graph neural network with high accuracy in node classification problems. More information on GCN can be found in Section 3. We compare RGCN, MedianGCN, adversarial training, and our proposed method under three adversarial attacking methods; FGA, NETTACK, and RL-S2V. Three benchmark defense strategies (R-GCN, MedianGCN, and adversarial training) are used and we briefly summarize them as follows:

- R-GCN uses the attentional mechanism to assign lower attention to suspicious nodes in the process of graph neural network aggregation. According to R-GCN, when the variance of a group of data is large, it means the dispersion degree of this group of data is large, and also means that uncertainty of this group of data is stronger. Hence, a neural network needs to assign less attention to this set of data. In order to achieve such a thought, R-GCN represents the hidden layer in GCN as a Gaussian distribution and calculates the variance from it. When the variance is larger, the weight will be smaller to a node;
- MedianGCN [25] aims to find out why graph convolutional networks are vulnerable to attacks. Based on the breakdown point theory, they point out that the vulnerability of graph convolutional networks mainly comes from the unrobust aggregation functions. The author regards the convolution process of GCN as a mean aggregation and the process of an adversarial attack tends to increase the extreme value and make the mean calculation move away. This makes the aggregation result deviate from the correct result. In this defense, the authors use the median instead of the mean. Compared to the mean, the median only changes when more than half of the values are changed. Therefore, the robustness of using the median calculation is better than that of the mean calculation;
- Adversarial training [19] is a classic defense against an adversarial attack. The basic idea of graph adversarial training is to include adversarial samples into the training stage of the model, expand the training samples, and train the original data and adversarial samples together, so as to improve the robustness of the model.

### 5.3. Environments

We will use the accuracy of the graph node classification task to verify the effectiveness of the defense scheme indirectly. The process of the experiment is as follows:

1. Training a healthy GCN model as target model and use it to complete the nodes classification task as a benchmark (original results). All the results are evaluated by accuracy;
2. Using the target model to complete the nodes classification task under attack and get attacked-results;
3. Nodes classification task under attack when the defense methods are involved. We get the third results;
4. Analyzing and comparing the three groups of results.

All the experimental methods, including attacking and R-GCN algorithms, are provided by the Deeprobust [35] python runtime library, which contains most of the classic and advanced adversarial attack and defense algorithms for graph data. All of our new approaches are written in Python 3.8.0.

The target model is a two-layer GCN model that combines classification accuracy with minimal operating expenses. The hidden layer of GCN is set to 16. Simultaneously, we used 80% of the nodes in the dataset as training sets to train the GCN model. The remaining 20% is for the test set.

### 5.4. Experiment and Results

**Figure 8:**
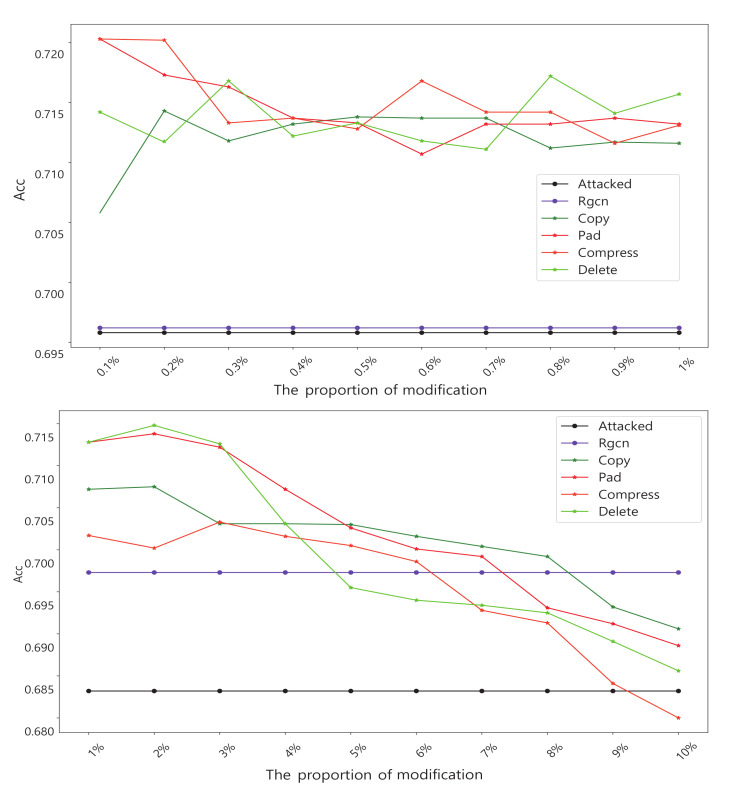
Experiments with a different transformation scale, We use 1% and 0.1% of the total number of nodes as steps for the experiment. The final result was measured by the accuracy (ACC) of the node classification task.

**Figure 9:**
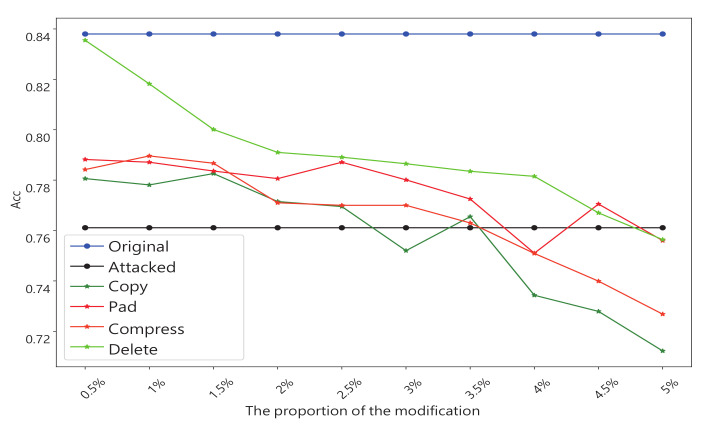
Experiments on poison attacks: An attacked model is trained as the benchmark. The final result is measured by the accuracy (ACC) of node classification task.

**Figure 10:**
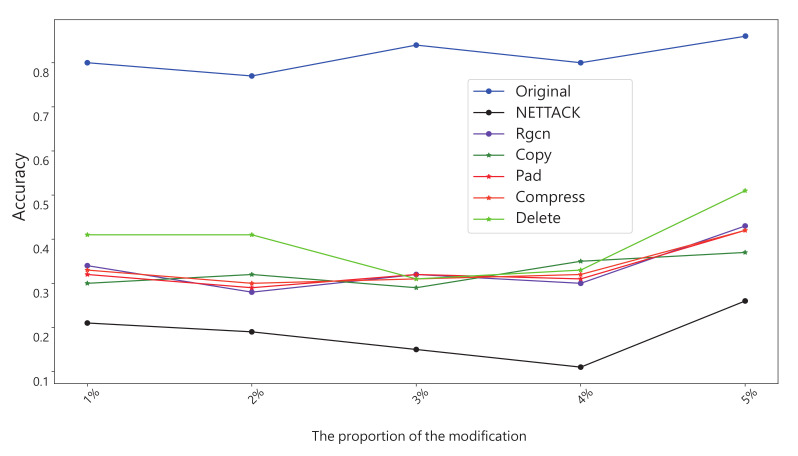
Experiment on local: 100 nodes were selected, focusing on the proportion of them that were correctly classified before and after an attack and before and after defense.

We constructed six series of experiments by introducing these graph data transformation methods into the GCN input layer as shown in Figure 2. Here we introduce four groups of experiments separately:

- Experiment 1: Basic defense experiment: We design the experiment with the setup in the previous subsection. The results are averaged over many experiments as shown in Table 3. It should be noted that this experiment was for evasion attacks, so we trained a GCN model as the target, which was restricted from being modified. This also means that we need to keep the number of nodes consistent with the original data, so we compressed the data before adding the extra nodes. When nodes compression is used alone, we modify it as 0 in the adjacency matrix instead of deleting the nodes directly.
- Experiment 2: Experiments with different transformation scale: In this experiment we focused on the amount of modification. We incrementally increase the number of changes by 1% of the total number of nodes. All experiments in this section will be targeted at NETTACK attacks. We increment the number of nodes by 0.1% in the first stage and 1% in the second stage. The experimental results are shown in Figure 8.
- Experiment 3: Experiments on run time: This set of experiments will intuitively demonstrate that our method has a significant time advantage over existing schemes. We chooses the Pubmed dataset, which is larger and more time-sensitive. Table 4 shows the results.
- Experiment 4: Defends against poisoning attacks: Although our method is originally designed to protect against evasion attacks, we also do experiments on poison attacks. The amount of each transformation that we make increases by 0.5% of the total number of nodes. The result is shown in Figure 9.
- Experiment 5: The above experiments evaluate the method from a global perspective. The fifth experiment focuses on specific nodes in the dataset before and after the defense. In this experiment, there are randomly selected some nodes as victims. At the same time, in the experiment, we ensure that these nodes will not be deleted in the process of defense. Finally, we looked at how correctly these victims were classified before and after attack and defense. The result is shown in Figure 10.
- Experiment 6: An experiment on run-time memory usage. In this experiment we used the Cora dataset. We let the experimental program interrupt before it calculates the AUC and gets the memory footprint of the python interpreter at this time. The experimental results are shown in Table 5.

### 5.5. Analysis and Discussion

In experiment 1, parameters consistent with real scenarios were used and repeated many times. It shows the general performance of our transformation method. The results in Table 2 show that it has good defense capability against gray box and black box attacks. The classification accuracy on transformed data can slightly surpass or is almost equal to the existing scheme. However, it is weak when defending against white box attacks. A white box attack is generally used to study model robustness and it is almost impossible for real-world attackers to have white box attack capability. Given the rarity of white-box attacks in real world scenarios, such shortcomings are grudgingly acceptable.

Experiment 2 focuses on the influence of the amount of transformations on the defense effect. Obviously, more changes to the raw data are not always better. These transformations are intended to destroy harmful structures, and when the amount of transformations is excessive, the damage to harmless structures will be greater. so, what amount is needed is a key question, and the results shown in Figure 8 for this experiment provide an answer. It shows that when the number of transformations exceeds 3% of the total number of nodes, the defense effect starts to decline rapidly. When the amount exceeds 10%, there is negative impact on node classification.

Experiment 3 visually demonstrates the time complexity of the algorithm. This experiment is mainly based on the Pubmed dataset because of its larger scale. It can be seen that our method already has an extremely significant time advantage over existing schemes when running on a dataset of about 20,000 nodes. At this point, the transformation method’s defense effect is almost equal to that of RGCN and increases the training epochs of RGCN will not achieve any further improvement.

Experiment 4 verifies that our method is also effective against a poison attack. We used a strategy similar to Experiment 2, gradually increasing the amount of transformation. The result is shown in Figure 9. It shows that the transformation method is also effective against poison attacks. However, it is best not to transform more than 3% nodes. The strategy of randomly deleting edges achieves the highest accuracy.

Experiment 5 focuses on the classification results of specific points before and after attack and defense. As shown in Figure 10, the classification accuracy of nodes after defense is increased by about 50%. This result also shows that our scheme is effective.

The results of experiment 6 show that our scheme has a slight advantage in memory usage. The average memory usage was calculated 3% lower than the existing method. Of course, the small scale of the Cora dataset we chose may be one reason for the small gap.

## 6. Conclusions

In this paper, a lightweight defending method with a graph transformation mechanism was proposed. Specifically, we designed four different graph transformation forms, including node-level and edge-level transformation. We also showed how to implement these transformation methods. In conclusion, our proposed defending method uses a graph transformation mechanism that relies on data instead of models or training, which is more practical in time or computational sensitive scenarios. The experiments on Cora and Pubmed show that the defense effect of this method is similar to existing schemes. In the face of black-box and grey-box attack approaches, the accuracy of the model can be restored to 80% while it only takes about 10% of the running time of existing solutions. Therefore, our method is more applicable in the case of finite computing power.

Although the transform method is effective and efficient, there are some issues regarding future work. Firstly, a more reliable way to select nodes or edges to be modified is needed. This will result in a more stable effect than the existing random selection method. The second is exploring the principle of the transformation method. In the future we will research these problems.

## Figures and Tables

**Table 1:** Comparison between the transformation strategy and the existing mainstream strategies.

Strategy	Need Any Extra Training?	Need to Replace the Existing Model?	Time Complexity
Adversarial training	Yes	Yes	O(n⌃2) (GCN directly adversarial training)
Robust neural network	Yes	Yes	O(n⌃2) * O(n) (r-GCN)
			O(n⌃2) (copy)
Transformation Method	No	No	O(n) (pad)
			O(n) (delete)
			O(nlog(n)) (compress)

**Table 2:** The main information of datasets.

Name	Node	Edge	Attribute	Label
Pubmed	19,717	44,338	500	3
Cora	2708	5429	1433	8

**Table 3:** Basic defense experiment.

Item	Original	Attacked	R-GCN	M-GCN ^1^	adv_train	Compress	Copy	Pad	Delete_Edge
NETTACK	0.846	0.718	0.788	0.790	0.805	0.791	0.794	0.796	0.795
FGA	0.846	0.643	0.651	/	0.723	0.642	0.643	0.642	0.647
RL-S2V	0.846	0.791	0.811	/	0.830	0.815	0.809	0.805	0.815

^1^ Note: As for the experiment of MedianGCN, we conducted it based on the source code provided by the author, so we did not carry out experiments for FGA and RL-S2V.

**Table 4:** Experiments on running time.

Item	Original	Attacked	R-GCN	M-GCN	adv_train	Copy	Pad	Delete_Edge	Compress
Acc	0.842	0.761	0.783	0.785	0.815	0.783	0.777	0.785	0.774
Time	/	/	85.701 s	74.267 s	74.486 s	2.563 s	1.660 s	0.196 s	1.471 s

**Table 5:** Memory usage comparison.

Method	Copy	Pad	Delete	Compress	Adversarial Training	r-GCN
Memory	2444.1 MB	2446.0 MB	2437.5 MB	2443.8 MB	2523.1 MB	2525.5 MB

## Data Availability

Not applicable.
